# Supplementary material for: Evolution of electronic states in n-type copper oxide superconductor via electric double layer gating
Source: Sci Rep. 2016 May 25;6:26642. doi: 10.1038/srep26642 (PMC4879525; doi:10.1038/srep26642)
Supplement: Supplementary Information [file srep26642-s2.pdf]

Supplementary information

Evolution of electronic states in n-type copper oxide superconductor via electric double layer gating

Kui Jin[1,2,3], Wei Hu[1], Beiyi Zhu[1], Dohun Kim[3,4], Jie Yuan[1], Yujie Sun[1], Tao Xiang[1,2], Michael S. Fuhrer[3,5], Ichiro Takeuchi[6], Richard. L. Greene[3]

[1]Beijing National Laboratory for Condensed Matter Physics, Institute of Physics, Chinese Academy of Sciences, Beijing 100190, China
[2]Collaborative Innovation Center of Quantum Matter, Beijing, 100190, China
[3]Center for Nanophysics and Advanced Materials and Department of Physics, University of Maryland, College Park, Maryland 20742, USA
[4]Department of Material Science and Engineering, Yonsei University, Seoul 120-749, Republic of Korea
[5] School of Physics, Monash University, Melbourne, Victoria 3800, Australia
[6]Department of Materials Science and Engineering, University of Maryland, College Park, Maryland 20742, USA

Supplementary Table 1| Charge carrier densities and conductivities of holes and electrons.

T(K)	V_G (V)	n_h ($\times 10^{25}$m^{-3})	n_e ($\times 10^{25}$m^{-3})	σ_h (mΩ^{-1}.cm^{-1})	σ_e (mΩ^{-1}.cm^{-1})
10	-2	6.72	6.22	1.64	1.58
	0	6.96	6.62	1.75	1.71
	+2	7.94	7.34	2.22	2.14
15	-2	6.07	5.75	1.65	1.60
	0	6.38	6.11	1.76	1.72
	+2	7.37	6.98	2.20	2.15
20	-2	6.96	6.28	1.66	1.57
	0	7.16	6.88	1.75	1.71
	+2	8.76	7.51	2.23	2.06

The data in the table are from the fits using two-band model.

Supplementary Note 1| Measurements

For the EDLT measurements, a four point probe Hall bar geometry (see Fig. 1a in the main text) was patterned by placing pre-patterned PDMS on the surface of PCCO thin film and performing dry etching with Ar plasma. The ohmic contact was formed by wire bonding Au wire with ductile Indium pallet. After mounting the sample on a cryostat PCB board, the samples were covered with the ionic liquid N, N-diethyl-N-methyl-N-(2-methoxyethyl) ammonium bis (triuoromethylsulphonyl) imide (DEME-TFSI, Kanto Corporation) and an adjacent Pt wire was used as a gate electrode. Ionic liquid application was performed inside a N_2 filled glove box and transferred to the measurement cryostat within 5 minutes to minimize electrochemical reaction of DEME-TFSI with molecules in the ambient atmosphere. Gate voltage was applied in situ at the temperature about 230 K inside the cryostat. Below 170 K the DEME-TFSI freezes and the longitudinal and transverse four probe resistance was measured as a function of temperature and perpendicular magnetic field. After each temperature sweep, gate voltage modulation was done by warming the sample up to 230 K and changing gate voltage.

Supplementary Note 2| Calculated quantities

The Fermi surfaces (FS) of both electrons and holes of PCCO thin film are cylindrical. The radius and the height of the FS are k_F and $2\pi/d$ respectively, where k_F is the Fermi wave length and d is the distance between two copper dioxide planes. Since the charge carriers are those particles enclosed by the FS, we can calculate k_F by the equation:

$$k_F = (2\pi n d)^{1/2} \qquad \text{(S1)}$$

where n is the charge carrier density obtained in our fitting and the value of d has been given in supplementary ref. 1. Then, using the equations:

$$k_F l = \frac{\hbar d \sigma}{e^2} \qquad \text{(S2)}$$

$$\xi_{GL} = \sqrt{\frac{\phi_0}{2\pi B_{c2}}} \qquad \text{(S3)}$$

$$\xi_{GL} = 0.855(\xi_{BCS} l)^{1/2} \qquad \text{(S4)}$$

$$\xi_{BCS} = \frac{\hbar v_F}{\pi \Delta_{max}} \qquad \text{(S5)}$$

$$\hbar k_F = m v_F \qquad \text{(S6)}$$

(supplementary ref. 2), we subsequently deduce the ratios of mean free paths l_e/l_h and effective masses m_h^*/m_e^* as shown in the main text. Here, σ is the conductivity of each band obtained from the two-band fitting.

Supplementary Note 3| Effective thickness

In general, we calculate the resistivity by taking into account the total thickness of the sample, i.e, $t \sim 10$ nm (7 unit cells). In our work, there is only one superconducting transition temperature. Meanwhile, the positive and negative electric fields result in

remarkable differences in the transport. Therefore, it is unlikely that there are multiple channels contributing to the electric transport, i.e, several unit cells at the top are tuned by the electric field but the other unit cells at the bottom still hold the T_c. It is naturally expected that the bottom several unit cells are insulating (namely, dead layer), whereas the top several unit cells can be tuned and contribute to electric transport. Thus, the effective thickness (t_{eff}) may be smaller than t when calculating the quantities of the electric transport. For instance, if $t_{eff} = \frac{t}{N}$,, then one can get $\rho_{xx}^{real} = \frac{\rho_{xx}}{N}, \rho_{xy}^{real} = \frac{\rho_{xy}}{N}, n^{real} = n \times N, \sigma^{real} = \sigma \times N, l^{real} = l \times N^{\frac{1}{2}}, m_{real}^{*} = m^{*} \times N$. Since the t_{eff} cannot be precisely estimated in this system, we still use t for all the calculations, which may cause an underestimation of the carrier concentrations, but not change the ratio of quantities of hole-band to electron-band (Fig. 4a, b, c). Our analyses and conclusions do not stand on the effective thickness.

Supplementary Note 4| Electrochemical reaction vs. electrostatic doping

We have shown in the main text that T_c is almost unaffected by the electrostatic voltage less than 2.5 V. Actually, we do observe a change of T_c if V_g > 2.5 V (see Fig. S1), where the change is caused by electrochemical reaction, rather than electrostatic tuning. We are interested in the intrinsic behavior of electronic states in electric fields, therefore, to avoid the influence from electrochemical reaction we only focus on the regime of lower gating voltage.

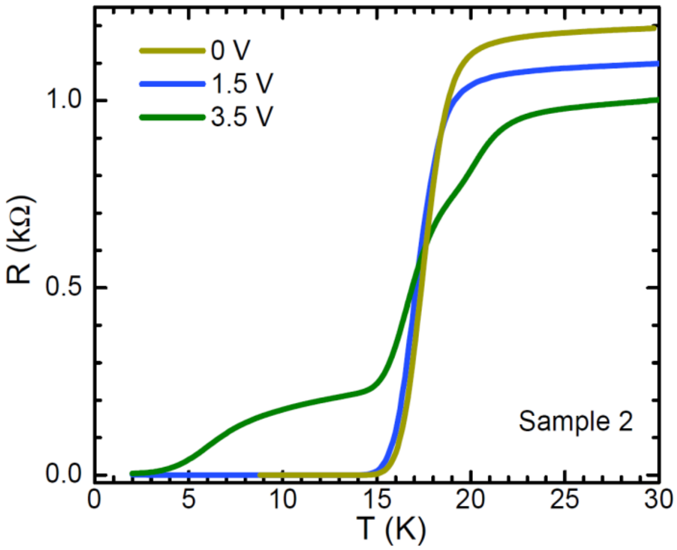

Figure S1 Temperature dependent resistance of sample 2 in electric fields of 0, 1.5 and 2.5 V. Both T_c^{onset} and T_c^{zero} are obviously tuned in 3.5 V, but the *R*-*T* curve exhibits two superconducting transitions which indicates an electrochemical reaction rather than electrostatic tuning.

Carton S1 Band structure evolution with chemical doping and electrostatic doping. The effective change transfer gap drops with chemical doping or electrostatic doping due to the Cu-O Coulomb repulsion and weakening on-site Coulomb interaction, which is in accordance with the two-band fitting results, where the carrier concentrations of electrons and holes are always enhanced (depressed) simultaneously in +2 V (-2 V).

Supplementary References

1. Armitage, N.P., Fournier, P. & Greene, R.L. Progress and perspectives on electron-doped cuprates. *Rev. Mod. Phys.* **82**, 2421-2487 (2010).
2. Dagan, Y., Beck, R. & Greene, R. Dirty superconductivity in the electron-doped cuprate $Pr_{2-x}Ce_xCuO_{4-\delta}$: tunneling study. *Phys. Rev. Lett.* **99** (2007).
